# Examining microbial assemblage differences on plants preferred and avoided by threatened Hawaiian land snails to identify important food resources

**DOI:** 10.1371/journal.pone.0361108

**Published:** 2026-10-26

**Authors:** Nelia S. Perry, André R. O. Cavalcanti, Kenneth A. Hayes, Norine W. Yeung, David R. Sischo, Samantha H. Shizuru, Adrienne K. Antonsen, Meagan L. Haubner, Ally N. Ah Cook, Charlton E. Hee, Mandilee M. Y. Hill, Constantine T. Krenteras, Taylor M. B. Maruno, Riley H. Nakasone, Hope K. Rosenbush, Sidney M. Stiefel, August J. Sullivan, Wallace M. Meyer III

**Affiliations:** 1 Molecular Biology Department, Pomona College, Claremont, California, United States of America; 2 Plant and Environmental Protection Sciences Department, University of Hawaii at Mānoa, Honolulu, Hawaiʻi, United States of America; 3 National Museum of Natural History, Smithsonian Institution, Washington, District of Columbia, United States of America; 4 Bishop Museum, Malacology, Honolulu, Hawaiʻi, United States of America; 5 Hawaiʻi Department of Land and Natural Resources, Honolulu, Hawaiʻi, United States of America; 6 Bishop Museum, Pacific Center for Molecular Biodiversity, Honolulu, Hawaiʻi, United States of America; 7 Department of Integrative Biology, Harvard College, Cambridge, Massachusetts, United States of America; 8 Phillips Academy, Andover, Massachusetts, United States of America; 9 Kamehameha Schools, Honolulu, Hawaiʻi, United States of America; 10 Biology Department, Pomona College, Claremont, California, United States of America; University of Salento: Universita del Salento, ITALY

## Abstract

The Hawaiian Islands, an isolated oceanic archipelago in the Pacific, had one of the world’s most spectacular land snail radiations. Unfortunately, only 35% of the 759 Hawaiian snail species remain, with >100 species threatened with imminent extinction. Though many extant Hawaiian snails are arboreal and are known to eat microbes on the surface of plants, our current understanding of which microbes compose arboreal Hawaiian snails’ diets remains rudimentary. Here, we examined microbial assemblages (bacterial and fungal) on plants previously identified as preferred and avoided by a diverse assemblage of arboreal snails at three sites in wet and mesic forests on O‘ahu, Hawai‘i, USA, to identify microbes that may form the foundation of Hawaiian arboreal snails’ diets. At Mt. Ka‘ala, we also investigated if microbial assemblages change across years. Across sites, microbial assemblages differed on preferred and avoided plants. While differences across sites can complicate the process of identifying key microbes, microbes with elevated abundances on preferred plants across sites are likely important food resources. Using this approach, we identified bacterial and fungal OTUs that we hypothesize form important components of Hawaiian arboreal snails’ diets. While we found that microbial assemblages differed among years at Mt. Ka‘ala, differences between preferred and avoided plants remained consistent across years. Expanding a snail plant preference–microbiome framework across multiple forest types is a crucial step in understanding arboreal snail diets as microbial taxa enriched on preferred plants across sites may be meaningful components of snail-associated microbial resources. Microbial taxa enriched on preferred plants are not presented here as confirmed dietary components, but as experimentally tractable candidates that should be further evaluated in laboratory trials. This information is foundational to improving captive rearing of critically endangered snails and informing habitat quality assessments for the many threatened Hawaiian snails.

## Introduction

Oceanic islands support disproportionately high levels of terrestrial molluscan diversity and endemism, and the Pacific Islands are home to more than 6,000 land snail species with most being single island/archipelago endemics [1–4]. The Hawaiian Islands once harbored at least 759 species with > 99% endemic to the archipelago [4,5]. Today, fewer than 35% of Hawaiian land snail species remain [4], with many persisting only as small, highly managed populations. Recent declines in populations across Hawai‘i have led experts to estimate that 100 or more Hawaiian land snail species will go extinct in the next decade without effective conservation efforts [6–8].

Unfortunately, despite decades of conservation intervention, progress has been constrained by limited ecological knowledge, particularly regarding diet and habitat quality for arboreal snails [4,9–12]. Because the Hawaiian land snail fauna is exceptionally diverse and many extant species continue to experience reductions in their range and population sizes, informed conservation strategies are urgently needed [4,7,8,13–15]. One challenge that has eluded conservation practitioners has been identifying important food resources for Hawaiian arboreal snails [16–21].

Hawaiian arboreal snails do not consume plant tissue directly. Instead, they graze bacterial and fungal assemblages inhabiting leaf surfaces and associated plant microhabitats [16,18]. These epiphytic microbial communities are diverse, spatially structured, and potentially sensitive to environmental conditions, making direct identification of dietary components challenging [16–21]. Previous to our work, studies employing metagenomic techniques in an effort to identify the diets of native snails have: (1) characterized the microbial assemblages on plants native snails are found on, (2) compared microbial assemblages on leaves to those in fecal and gut samples, and/or (3) examined differences in microbial assemblages in mesocosms with and without snails present [17–20]. As only small differences in microbial assemblages were found between leaves and snail feces and between mesocosms with and without snails, the conclusions from most of these studies are that snails are generalist grazers, or ‘not picky eaters’ [17–20]. However, these experiments have solely examined assemblages on plants snails are found on. Our research has found that snails exhibit consistent preferences for a small subset of plant species across sites within and among islands [21–25], and bacterial and fungal assemblages differ on plants snails prefer and avoid [21]. Consequently, arboreal snails may be selecting plants based on their epiphytic microbial assemblages suggesting that snail feeding behaviors are more nuanced than the classification of generalist grazers [21].

Meyer et al. [21] proposed a framework integrating plant preference surveys with comparative analyses of microbial assemblages on preferred and avoided plants. Applied at a single site (Mt. Ka‘ala Natural Area Reserve, Oʻahu), this approach revealed distinct bacterial and fungal assemblages associated with preferred plants, suggesting that snail plant selection may be mediated by microbial composition rather than plant identity alone. Whether this pattern generalizes across sites, forest types, and years remained unknown. Recently, expanded efforts to better understand plant preferences of arboreal snails on O‘ahu have included reexamining snail preferences at Mt. Ka‘ala and assessing plant preferences at eight additional sites across the island [25]. While each site harbors unique assemblages of snail species, composed of individuals from many diverse families, and distinct plant compositions [25], concordant patterns in snail plant preferences across sites and islands highlight that certain plant species may be critically important for a variety of arboreal snail species [21–25]. Because we see concordant patterns in snail plant preferences among a variety of snail species within and among snail families [22–25], these studies are likely to inform plant preferences of endangered snail species in which we have less data. Unpublished work by authors of this manuscript and Price et al. [20] confirm that endangered *Achatinella* snails use the plant species identified as being important for Hawaiian land snails by Meyer et al. [25]. In addition, using surrogate snail species to inform conservation of endangered snail species is common [e.g., 18,26,27]. Reassessment of snail plant preferences in Mt. Ka‘ala a decade after our initial plant preference study at that site found that snail preferences remained consistent. Thus, these short-term, but targeted, person-hour intensive, surveys yield critical ecological information that can be informative for understanding snail plant preferences over long time periods.

Using these plant preference data, we expand on Meyer et al.’s [21] approach, extending this framework across three wet and montane mesic forest sites on Oʻahu and evaluate temporal consistency using multi-year data from Mt. Kaʻala. Specifically, we ask: (1) do bacterial and fungal assemblages differ between plants preferred and avoided by arboreal snails across sites, (2) how strongly site-level differences structure these assemblages relative to preference category, (3) are observed patterns at Mt. Kaʻala stable across years, and (4) are particular microbial taxa consistently enriched on preferred plants across sites. By addressing these questions, we aim to identify candidate microbial components of arboreal snail diets and provide an empirical foundation for improving captive rearing, habitat restoration, and conservation monitoring efforts.

## Materials and methods

### Study sites

Of the nine sites surveyed by Meyer et al. [25] to assess plants preferred by snails on O‘ahu, three sites (Table 1) were selected based on the following parameters: (1) sites contained a high number (> 500) of native arboreal snails in plant preference surveys (see [25]); (2) sites were primarily composed of native plants to be able to assess feeding behaviors of snails on native plants [23,25]; and (3) sites span both mountain ranges on O‘ahu allowing for initial insights into how microbial assemblages may differ across mountain ranges and, more importantly, if patterns between preferred and avoided plants persist between mountain ranges. Two sites were in the Wai‘anae Mountains, while another was in the Ko‘olau Mountain Range (Table 1). Mt. Ka‘ala and Makiki are wet forests while Palikea is a montane mesic forest. We defined wet forests as those receiving an average annual rainfall above 2,000 mm and mesic forests as having an annual rainfall below 2,000 mm [29]. Since the microbial assemblages on preferred and avoided plants had been previously sampled at Mt. Ka‘ala in July 2018 and November 2020 [21], our survey of Mt. Ka‘ala in June 2023 allowed us to further explore if microbial assemblages differ across years and seasons and if differences between plants preferred and avoided by snails persist. In addition, snail preferences in Mt. Ka‘ala were examined twice, once in June 2013 and again in June 2023 [23,25]. Snail preferences for plants were consistent across decades with 5 of 5 and 5 of 6 of the abundant snail species (species with > 30 individual snails at a site) preferring *Hydrangea arguta* and *Ilex anomala* in 2013 and 2023, respectively [23,25]. In Hawai‘i there are two seasons: a dry summer from May to October and a wet winter from November to April [29]. Samples from 2018 (July, dry season) and 2020 (November, wet) were collected by Meyer et al. [21] and were only used in our analysis comparing microbial assemblages across years/seasons.

**Table 1:** Habitat classification, mean annual rainfall, sampling dates, and number of 5 x 5 m plots surveyed for each site on O‘ahu.

Site Factor	Mt. Ka‘ala Natural Area Reserve (Mt. Ka‘ala)	Honouliuli Forest Reserve (Palikea)	Pu‘u ʻŌhiʻa (Makiki)
**Habitat Classification** ^a^	Wet forest	Montane mesic forest	Wet forest
**Mountain Range**	Wai‘anae Mountain Range	Wai‘anae Mountain Range	Ko‘olau Mountain Range
**Mean Annual Rainfall (mm)** ^**b**^	2015.8	1166.6	2436.3
**Sampling Date**	June 2023	July 2023	July 2023
**Number Plots Surveyed**	8	6	6

^a^ Wet forests have an annual rainfall above 2,000 millimeters and mesic forests have an annual rainfall below 2,000 millimeters.

^b^ Giambelluca et al. [28].

### Experimental approach: Plant selection and microbial sampling

We employ Meyer et al.’s [21] approach, which first determines plant preferences of native Hawaiian arboreal snails at sites and then examines if microbial assemblages differ between plants preferred and avoided by Hawaiian arboreal snail species. Plant preferences of native Hawaiian snails were explored by Meyer et al. [25]. Inclusion in this study as either a plant preferred or avoided by snails was determined using a subset of criteria and discussing categorical inclusion or exclusion (Table 2). Avoided plants were plant species that were never preferred (Jacobs’ Index < 0) by any common snail species (abundance > 30 individuals) at that site. The one exception is *Cibotium chamissoi* at Makiki which had one positive Jacobs’ Index score of 0.08. It was included as this species was strongly avoided by the other two abundant snail species (Jacob’s Index < - 0.8) and because this species had never met the criteria (Jacob’s Index > 0.25) to be considered a preferred plant species in any previous preference study despite being abundant at most sites surveyed [22–25]. Meyer et al. [25] did not provide variance measures for reported Jacob’s indices, but focused on concordant patterns across snail species and sites. Plants species were prioritized for inclusion if they had high percent cover and were found in multiple plots at a site enhancing the opportunity for snails to utilize that plant species. Plant designation as preferred or avoided was entirely based on preferences of the snail species at that site. Of the 12 plant species categorized as avoided in this study, only two were on Meyer et al.’s [25] list of potentially important plant species for Hawaiian arboreal snails which identifies plant species that were preferred by at least one common snail species (> 30 individuals) at the nine sites they surveyed on O‘ahu: *Metrosideros polymorpha* var. *glaberrima* (preferred in 13% (2/15) of the observations) and *Clidemia hirta* (15%; 2/13). Neither of these plants were included in the list of 16 plant species that were preferred more often than they were avoided across the nine sites on O‘ahu [25]. In contrast, preferred plant species had to be preferred by at least 50% of the common snail species at a site. However, we prioritized plants that were preferred by a majority (> 50%) of the common snail species. In addition, we also considered individuals where there were strong preferences (Jacobs’ Indices > 0.25). While we considered relative cover of the plants in designating preferred plants, we gave more weight to plant species that were preferred by more snail species, i.e., having more snail species prefer the plant at the site was a more important criterion. Consequently, some plant species were included as preferred plants here had relatively low cover. Authors (Perry and Meyer) independently created their lists and discussed categorical inclusion of each plant species prior to any molecular analyses. All plants species were identical on both Perry and Meyer’s lists, except for *Blechnum appendiculatum* which was included as a preferred species at Palikea after discussion, as the two most abundant snail species at that site had strong preferences for this plant species. While we recognize that other approaches could be used to categorize plants as preferred and avoided, our approach is conservative in determining if preferred and avoided plants across sites have different microbial assemblages. At our three sites, 12 snail taxa from four families (Achatinellidae, Euconulidae, Succineidae, and Pupillidae) had abundances that exceeded the 30 individual threshold and were considered common (Table 2). These four snail families are the most common and abundant extant arboreal snail families in Hawaiʻi [4] and had largely concordant plant preferences [25]. In addition, 6 of the snail taxa were from the Achatinellidae. Previous studies have used surrogate achatinellids to inform endangered Hawaiian tree snail (*Achatinella* spp.) conservation efforts [18,19,26,27].

**Table 2:** Plant samples used to compare microbial assemblages between preferred (P) and avoided (A) plant species at each site.

Site *Plant Species*	% Cover	P or A	No. Species P / A	Jacob’s Index Scores	No. Samples
Mt. Ka‘ala					
*Hydrangea arguta*	19.4	P	4/1	0.47^**3**^, 0.41^5^, 0.40^2^, 0.31^1^, −0.65^4^	8
*Ilex anomala*	6.6	P	4/1	0.85^5^, 0.82^2^, 0.75^1^ 0.61^3^, −1.0^4^	6
*Metrosideros polymorpha* var. *glaberrima*	51.0	A	0/5	−0.08^3^, −0.44^1^, −0.57^5^ −0.64^2^, −1.0^4^	7
*Cibotium glaucum*	25.3	A	0/5	−0.53^2^, −0.80^1^ −1.0^3^, −1.0^4^, −1.0^5^	7
*Cibotium chamissoi*	5.9	A	0/5	−0.81^1^, −1.0^2^, −1.0^3^, −1.0^4^, −1.0^5^	3
Palikea					
*Coprosma longifolia*	3.1	P	4/0	0.67^8^, 0.64^9^, 0.37^7^ 0.12^6^	2
*Kadua affinis*	3.1	P	4/0	0.65^6^, 0.64^9^, 0.55^8^, 0.01^7^	2
*Psychotria mariniana*	0.8	P	4/0	0.97^6^, 0.95^8^, 0.92^9^, 0.50^7^	1
*Touchardia oahuensis*	3.4	P	3/1	0.96^8^, 0.46^6^, 0.16^9^ −0.62^7^	3
*Asplenium acuminatum*	2.4	P	3/1	0.71^6^, 0.65^7^, 0.11^8^, −1.0^9^	1
Blechnum appendiculatum*	14.7	P	2/2	0.64^6^, 0.21^7^, −0.68^8^, −1.0^9^	7
*Diplazium sandwichianum*	15.9	A	0/4	−0.42^7^, −0.57^9^, −.061^6^, −1.0^8^	6
Christella parasitica*	15.1	A	0/4	−0.71^6^, −.072^7^, −1.0^8^, −1.0^9^	7
*Pipturus albidus*	10.5	A	0/4	−0.24^7^, −0.94^6^, −1.0^8^, −1.0^9^	7
*Cibotium chamissoi*	7.1	A	0/4	−0.29^7^, −0.69^6^, −1.0^8^, −1.0^9^	5
Passiflora suberosa*	4.0	A	0/4	−0.15^8^, −0.47^7^, −0.73^6^, −1.0^9^	5
Makiki					
Hedychium coronarium*	8.8	P	3/0	0.72^1^, 0.37^2^, 0.19^3^	5
*Antidesma platyphyllum*	2.3	P	3/0	0.89^1^, 0.81^3^, 0.53^2^	2
*Nephrolepis exaltata* subsp. *hawaiiensis*	1.4	P	3/0	0.84^2^, 0.84^3^, 0.32^1^	1
*Asplenium excisum*	1.4	P	3/0	0.50^11^, 0.23^10^, 0.14^12^	2
*Pritchardia* sp.	0.4	P	3/0	0.85^11^, 0.78^12^, 0.49^10^	1
*Ceodes brunoniana*	0.2	P	3/0	0.95^10^, 0.95^11^, 0.92^12^	1
*Touchardia oahuensis*	0.2	P	3/0	0.81^11^, 0.78^12^, 0.49^10^	1
*Psychotria mariniana*	4.9	P	2/1	0.50^10^, 0.31^12^, −0.65^11^	2
*Psydrax odorata*	0.2	P	2/1	0.92^11^, 0.49^10^, −1.0^12^	1
Oplismenus hirtellus*	43.1	A	0/3	−0.56^12^, −0.78^10^, −0.78^11^	5
Setaria palmifolia*	9.7	A	0/3	−0.66^11^, 0.70^12^, −1.0^10^	5
*Cibotium menziesii*	9.3	A	0/3	−1.0^10^, −1.0^11^, −1.0^12^	1
Clidemia hirta*	6.5	A	0/3	−0.18^11^, −0.28^12^, −0.62^10^	6
Citharexylum caudatum*	4.2	A	0/3	−0.40^12^, −1.0^10^, −1.0^11^	1
*Cibotium glaucum*	0.4	A	0/3	−1.0^10^, −1.0^11^, −1.0^12^	1
*Cibotium chamissoi*	22.9	A	1/2	0.08^11^, −0.081^10^, −0.87^12^	5

^1^*Elasmias luakahaense* (302 individuals), Family: Achatinellidae; ^2^*Philonesia hartmanni* (67), Euconulidae; ^3^*Succinea* sp. (46) Succineidae; ^4^*Pronesopupa acanthinula* (42), Pupillidae; ^5^*Lamellidea* cf. *polygnampta* (33), Achatinellidae; ^6^*Tornatellides* spp. (531), Achatinellidae; ^7^*Philonesia* spp. (229), Euconulidae; ^8^*Elasmias* cf *fuscum obtusum* (124), Achatinellidae; ^9^*Pronesopupa* spp. (40), Pupillidae; ^10^*Elasmias* sp. (868), Achatinellidae; ^11^*Tornatellides* spp. (516), Achatinellidae; ^12^*Pronesopupa hystricella* (106), Pupillidae.

*Non-native plants. In addition to categorical designations (P and A), we include summary plant preference data from Meyer et al. [25], including: the Jacobs’ selectivity score for each common snail species (> 30 individuals at a site). Jacobs’ selectivity scores range from 1 to −1, with scores above zero indicating preference and scores below zero indicating avoidance. Meyer et al. [25] did not calculate variances for Jacobs’ indices. We provide the number of genetic samples from each plant species used in this study, which corresponds to the number of plots that plant species was found in at each site. Snail species and family identity and abundances are provided in footnotes.

Microbial sampling took place concurrently with the plant preferences assessment reported in Meyer et al. [25]. Microbial sampling consisted of swabbing all plant species in every plot examined using sterile cotton swabs dipped in Longmire’s solution to obtain the microbiome community from stems, and leaves (top and bottom) and node of each plant. An individual swab was used for the stem, leaf, and node sections of each plant sampled. All three swabs compose one sample which integrates microbes from all three plant parts. While this limits our ability to assess the potential importance of plant part [30], these integrated samples allow us to explore differences in microbial assemblages among plant species. In addition, using individual swabs for different plant parts should help to standardize abundances across plant parts in our integrated sample. All three cotton swabs were placed in sterile 1.5 mL microcentrifuge tubes and stored on cold blocks (−20 °C) for transport back to the Bernice Pauahi Bishop Museum. All samples were stored in an ultracold (−80 °C) freezer prior to DNA extraction.

### DNA Extraction and Sequencing

Genomic DNA was extracted using the Macherey-Nagel NucleoSpin® Stool Kit following the manufacturer’s protocol with modifications for cotton swabs. Swabs were stored in a cold block after removal from the −80 °C freezer. The outer part of the swab containing the sample was cut away using sterile scissors, being careful to minimize excess cotton material from each swab. All three samples from each plant were combined in a single bead tube with 750 µl lysis buffer, allowing for all swab material to be fully covered, and 300 µg Proteinase-K was added for overnight digestion at 65 °C. Eluted DNA was quantified using the Qubit® 3.0 fluorometer (Thermo Fisher Scientific, USA) and stored at −20 °C prior to Illumina short-read sequencing at Novogene. Bacterial 16S DNA was amplified using primers 515F-Y and 926R [31], and fungal ITS2 regions were amplified using primers ITS1-F [32] and ITS2 [33].

Both 16S and ITS fragments were amplified using the Phusion® High-Fidelity PCR Master Mix (New England Biolabs) with the following cycling conditions: an initial denaturation of 30 seconds at 98 °C, followed by 30 cycles of 10 seconds at 98 °C, 30 seconds at 58 °C and 30 seconds per kilobase at 72 °C, and a final elongation step of 10 minutes at 72 °C. PCR product quantity and quality were determined via visualization on a 2% agarose gel. Following this verification, PCR products were mixed in equimolar ratios and purified with Qiagen Gel Extraction Kit (Qiagen, Germany). Finally, sequencing libraries were generated using NEBNext® UltraTM DNA Library Pre Kit for Illumina, following manufacturer ‘s instructions. The library quality was assessed on the Qubit® 2.0 Fluorometer (Thermo Fisher Scientific, USA) and Agilent Bioanalyzer 2100 system. The library was sequenced on the MiSeq Illumina platform and 250 bp paired-end reads were generated. All sequence reads are available on NCBI’s GenBank (Bioproject: PRJNA880325).

### Bioinformatics

All raw reads were processed using QIIME2 [34] version 2026.2.0. For all reads, we used the Cutadapt plugin [35] to remove primers from reads and to filter out reads without primer sequences. All reads were denoised using DADA2 [36] and unique amplicon sequence variants (ASVs) and the corresponding counts per sample were identified. ASVs that appeared in a single sample or with a total count of less than 0.1% of the average read depth of the samples were removed. For inter-year analyses, the data from 2018 and 2020 (see [21]) is also available in GenBank (BioProject: PRJNA880325) and was re-analyzed separately using the same procedure as the current data. The two bacterial and two fungal ASV tables (one for 2018/2020 and one for 2023) were merged using QIIME2’s feature-table plugin, resulting in one bacterial and one fungal ASV table.

Although amplicon sequence variants (ASVs) provide high-resolution representations of microbial diversity [37], we clustered ASVs into operational taxonomic units (OTUs) at 97% sequence similarity for downstream analyses using the “cluster-features-de-novo” option of QIIME’s VSearch plugin [38]. This decision was made as OTUs are more practical targets for culturing and experimental manipulation in feeding trials, where isolating and maintaining strains corresponding to individual ASVs is often infeasible [39,40], and grouping closely related variants likely better reflects the scale at which arboreal snails perceive and respond to microbial resources, as fine-scale nucleotide differences do not necessarily correspond to meaningful functional or ecological differences [41,42]. However, OTU clustering reduces analytical sensitivity relative to ASV-based approaches and may underestimate subtle differences among sites or sampling periods [37,43]. It is important to note that Meyer et al. [21] based their analysis on ASVs.

Taxonomic designations for each OTU were determined using the VSearch option of the q2-feature-classifier plugin [38,44] and bacterial and fungal reads were mapped against SILVA v138.0 [45] and UNITE v9.0 [46] databases, respectively. Only OTUs matching bacterial or fungal sequences were retained.

For the site analysis, we removed all samples from 2018/2020 and rarefied the bacterial and fungal samples from 2023 based on the number of reads in the sample with the fewest reads. The alpha and beta diversity measures were then calculated using the diversity plugin for QIIME2.

For each sample, we calculated the number of different OTUs (α-richness), diversity (Shannon Entropy), and evenness (Pielou Evenness index). To estimate the differences in diversity between samples (β-diversity), we used the Bray-Curtis index.

For the interannual analysis, we only used samples from Kaʻala [21], we removed the *Clermontia* sp. samples as these were not collected in 2023. Using only these samples, we rarefied the bacterial and fungal samples based on the number of reads in the sample with the fewest reads, and calculated the Bray-Curtis index between samples using QIIME2’s diversity plug-in.

All the QIIME2 commands used in the sequence analyses are available as a repository at GitHub: https://github.com/aroc110/Perry_et_al_2026. A sample by OTU matrices for bacteria and fungi are provided in S1.

### Statistical analysis

For all analyses, alpha and beta diversity calculations were determined in QIIME2 and uploaded into PRIMER-E with the PERMANOVA+ add-on [47]. To test if microbial compositions differed between preferred and avoided plants and across sites, we ran two, two-factor, PERMANOVA tests (one for bacteria and one for fungi). For each PERMANOVA, Bray-Curtis distance matrices were used. Site and preference category (preferred and avoided) were fixed factors. Following a significance test for site or site by preference interaction, we ran pairwise permutation-based t-tests to assess which sites had different microbial assemblages. After identifying significant preference effects, we ran contrast tests to confirm that preferred and avoided plants harbored different microbial assemblages at each site. To visualize relationships among samples according to the composition and abundance of microbial species we created two MDS plots combining all samples from all three sites for both bacteria and fungi.

To test if microbial compositions differ among years and seasons at Mt. Ka‘ala, we ran two, three-factor PERMANOVA tests (one for bacteria and one for fungi) using year (July 2018, November 2020, and June 2023) and preference category (preferred and avoided) as fixed factors. As samples from all years were collected from the same eight 5 x 5 m plots, we incorporated plot as a random factor [21]. While samples from each plant species were taken from the same eight plots during each sampling period, there were at least two years between each sample which is ample time for microbial assemblages to change as microbes have short generation times. In addition, there are multiple individuals of most plant species in a plot, making it unlikely that samples were taken from the same individual. Consequently, we made the assumption that samples from the same plant species among years represent independent replicates. For this analysis, we excluded *Clermontia* sp. which was sampled by Meyer et al. [21] but not sampled in June 2023. Similarly, we included three *Metrosideros polymorpha* var. *polymorpha* samples, as variety-level identification was not incorporated in previous studies [21]. While Meyer et al. [21] used ASVs for their PERMANOVA tests, consistent with all our other analysis, OTUs were used for interannual analyses, which could produce different results between July 2018 and November 2020 that were previously compared at the ASV level. Following significant year, or year by preference interactions, we ran pairwise permutation-based t-tests to assess which years had different microbial assemblages. We constructed Multi-dimensional scaling (MDS) plots for both bacteria and fungi to visualize differences in microbial assemblages between preferred and avoided plants across the three collection periods that spanned five years and had samples collected in the two Hawaiian seasons.

To test if bacterial and fungal OTU α-richness, diversity (Shannon), and evenness (Pielou) differed between preferred and avoided plant species among the three sites, we ran six univariate PERMANOVAs. Matrices for each metric (α-richness, diversity, and evenness) were created using the Euclidean similarity index. Following significant tests for site or site by preference interactions, we ran pairwise permutation-based t-tests to assess which sites differed for each metric and to determine at which sites these metrics were elevated or reduced on preferred plants. For all pairwise t-tests comparing differences across sites, we used a Bonferroni correction to account for multiple testing (α-values = 0.0167).

### OTU Selection

To identify OTUs that may form the foundation of arboreal snail diet, we applied two approaches. First, we applied several filtering criteria to the dataset and ranked OTUs according to these criteria: We first removed any OTUs that had a relative abundance below 0.01% at all sites. We then calculated a preference ratio for the remaining OTUs at each site by using the equation: *(P − A)/(P + A)*, where *P* represents the relative abundance of the OTU on preferred plants and *A* represents the relative abundance on avoided plants. This provides values ranging from −1–1 for each site, where −1 indicates the OTU is only present on avoided plants, 1 represents the OTU is present only on preferred plants, and 0 indicates that the OTU was present equally on preferred and avoided plants. We did not calculate variance measures for these preference scores. Instead, we focused on concordance across sites (e.g., which microbes consistently had elevated abundance on preferred plants across sites) and gave additional weight to microbes with high relative abundances. To do this, we removed OTUs that had negative values, i.e., had a higher abundance on avoided plants, at one or more sites. We then removed OTUs with a preference ratio below 0.25 at two or more sites. For the remaining OTUs, we averaged the percent abundance of the OTU on preferred plants at all three sites and ranked the OTUs from highest to lowest on preferred plants, as we expect that higher relative abundances correspond to greater impacts on snail plant preferences. Second, we examined the 10 most abundant OTU groups by relative abundance on preferred plant species. We did this to better visualize the distribution of dominant OTUs across sites and between plants snails prefer and avoid.

## Results

### Microbial compositions among sites

Bacterial (F_1_ = 2.27, P = 0.004) and fungal (F_1_ = 2.34, P = 0.0001) assemblages differed between preferred and avoided plants (Fig 1, Table 3). For bacteria (F_2_ = 20.7, P = 0.0001) and fungi (F_2_ = 6.68, P = 0.0001), assemblages also differed among sites (Fig 1), and site by preference interactions were significant for both bacteria (F_2_ = 2.58, P = 0.0001) and fungi (F_2_ = 2.27, P = 0.0001). For bacteria, contrast tests indicate that bacterial assemblages differed on preferred and avoided plants at Mt. Ka‘ala and Palikea, but not at Makiki (Table 3). Fungal assemblages differed on preferred and avoided plants at all three sites (Table 3).

**Fig 1:**
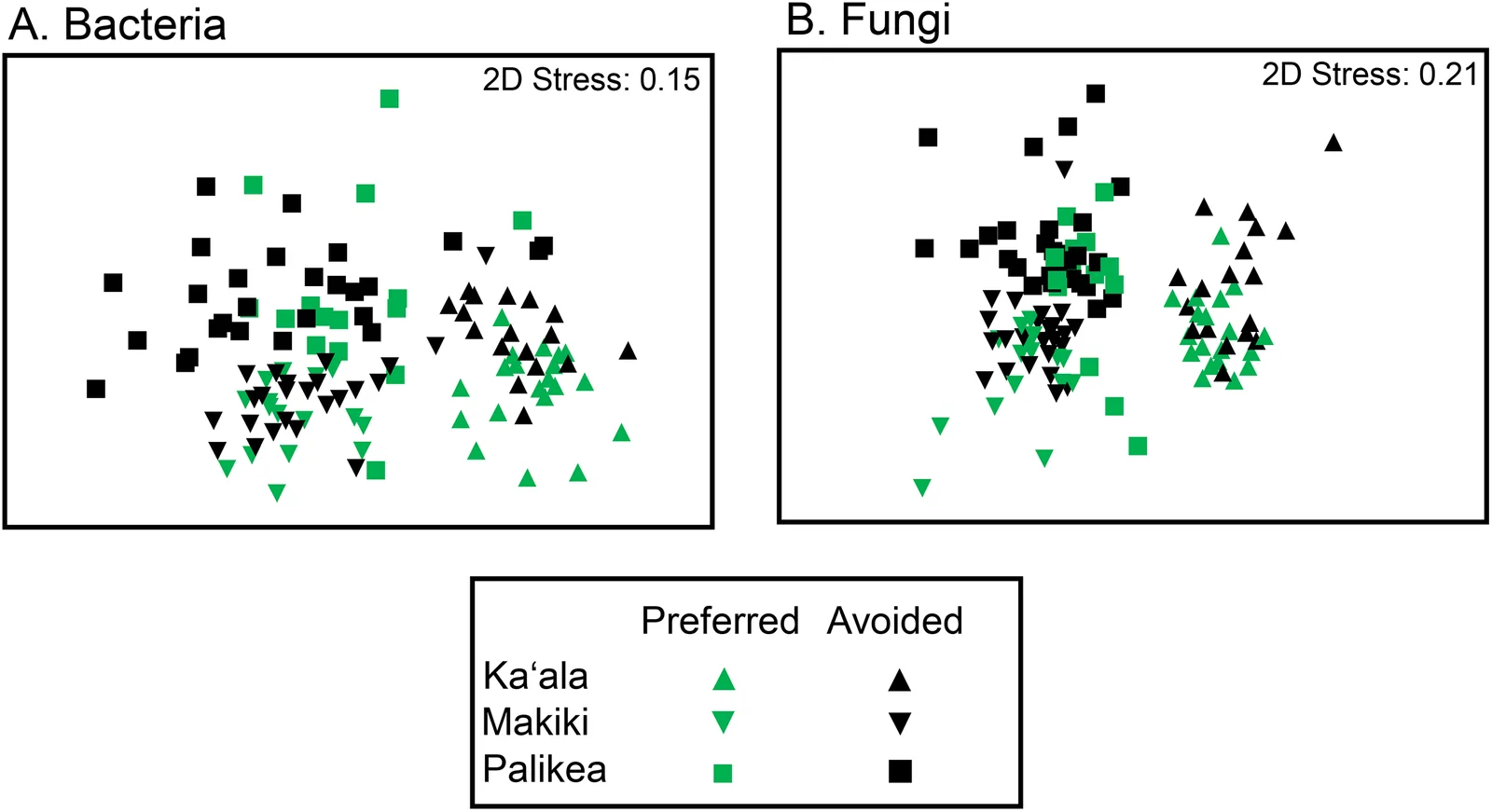
MDS ordination showing the relationship among sites (Mt. Ka‘ala, Makiki, and Palikea) and samples on plants preferred and avoided plants snails using the relative abundances of bacterial and fungal OTUs. Similarity was calculated using Bray-Curtis distances. Samples that are closer together are more similar in microbial compositions. All samples here were collected in 2023.

**Table 3:** Pairwise comparisons and contrast tests for assemblages on preferred and avoided plants, assemblages by site, and assemblages by year/season. Corrected α-values were 0.0167.

Pairwise Comparisons	Bacteria		Fungi	
	t	P_(perm)_	t	P_(perm)_
Preferred vs Avoided				
Mt. Ka‘ala	2.00	0.0001	1.83	0.0001
Makiki	1.10	0.18	1.32	0.0005
Palikea	1.56	0.0028	1.37	0.0005
Sites				
Mt. Ka‘ala vs Makiki	5.86	0.0001	2.88	0.0001
Mt. Ka‘ala vs Palikea	4.59	0.0001	2.67	0.0001
Makiki vs Palikea	3.11	0.0001	2.17	0.0001
Between Years at Mt. Ka‘ala				
2018 vs 2020	1.03	0.49	1.11	0.39
2018 vs 2023	1.47	0.21	1.58	0.13
2020 vs 2023	2.28	0.0034	2.26	0.0037

### Microbial compositions across years/seasons

At Mt. Ka‘ala, bacterial and fungal assemblages differed across years (bacteria: F_1_ = 3.31, P = 0.0011; fungi: F_1_ = 3.32, P = 0.0003) and preference category (bacteria: F_1_ = 5.80, P = 0.0020; fungi: F_1_ = 3.50, P = 0.0042, Fig 2). There was no significant year by preference interaction for bacteria (F_2_ = 1.20, P = 0.28) or fungi (F_2_ = 1.30, P = 0.18). Bacterial (F_7_ = 0.98, P = 0.54) and fungal (F_7_ = 0.98, P = 0.59) assemblages did not differ among plots. Pairwise comparisons revealed that while assemblages did not differ for bacterial and fungal compositions between July 2018 and November 2020, the June 2023 bacterial and fungal assemblages differed from those from November 2020, but not July 2018 (Table 3).

**Fig 2:**
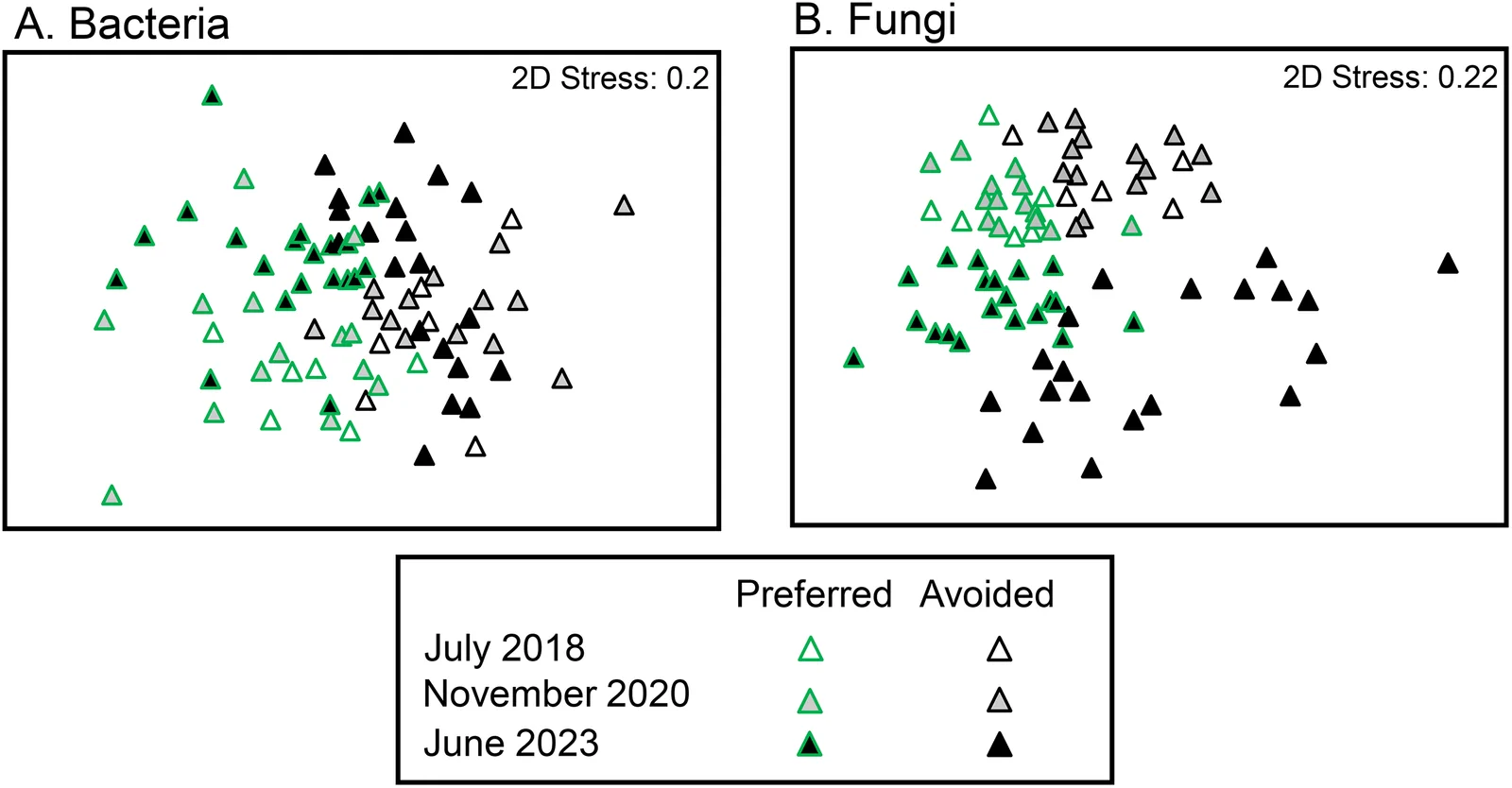
MDS ordination showing relationship between preferred and avoided plants in 2018, 2020, and 2023 at Mt. Ka‘ala according to the composition and relative abundance of bacterial and fungal OTUs on each plant. Similarity was determined using Bray-Curtis distances. Samples that are closer together have similar microbial compositions.

### ⍺-richness (OTUs), diversity (Shannon), and evenness (Pielou)

We found that bacterial diversity (F_1_ = 0.22, P = 0.63) and evenness (F_1_ = 2.06, P = 0.16) did not differ between preferred and avoided plants (Fig 3). However, bacterial α-richness did (F_1_ = 4.85, P = 0.028; Fig 3). Pairwise comparisons revealed bacterial α-richness did not differ between preferred and avoided plants at Makiki or Palikea, but α-richness was elevated on preferred plants at Mt. Ka‘ala (Fig 3). Additionally, α-richness (F_2_ = 6.18, P = 0.006), diversity (F_2_ = 7.44, P = 0.0006), and evenness (F_2_ = 6.88, P = 0.0007; Fig 3) differed among sites. Pairwise comparisons revealed that bacterial α-richness was similar between Makiki and Mt. Ka‘ala, but elevated at Makiki relative to Palikea. Bacterial diversity and evenness was elevated at Mt. Ka‘ala and Makiki compared to Palikea (Fig 3). There were no significant site by preference interactions for bacteria (α-richness: F_2_ = 0.85, P = 0.43; diversity: F_2_ = 0.60, P = 0.55; evenness: F_2_ = 0.79, P = 0.46).

**Fig 3:**
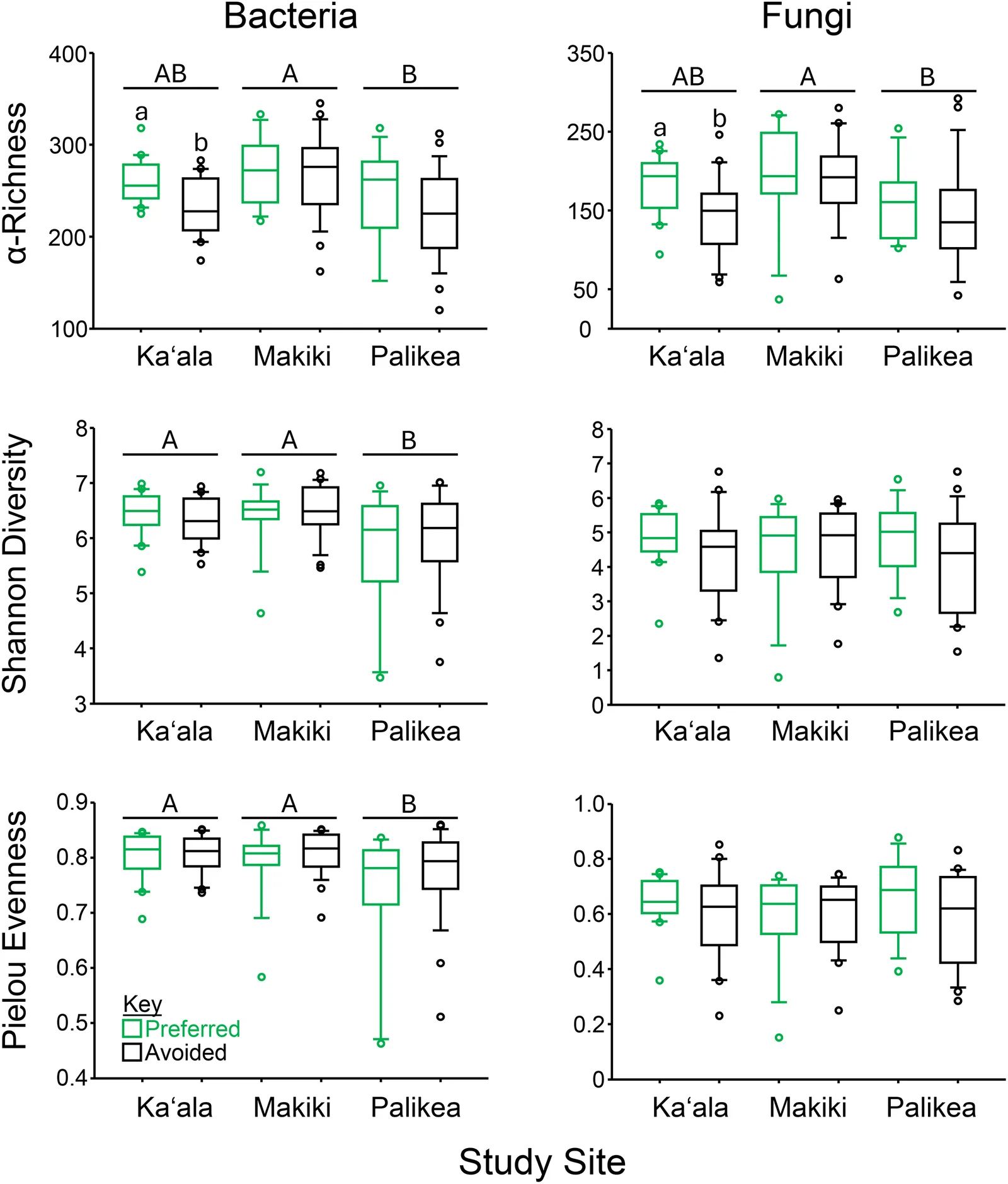
Bacterial and fungal OTU α-richness, diversity (Shannon), and evenness (Pielou) for preferred (green) and avoided (black) plants at Mt. Ka‘ala (K), Makiki (M), and Palikea (P). Upper case letters (A / B) with horizontal lines indicate differences between sites, lower case letters (a / b) indicate differences between preference categories. Samples collected in 2023.

For fungi, α-richness (F_1_ = 4.10, P = 0.045) differed between preferred and avoided plants, but diversity (F_1_ = 3.44, P = 0.069), or evenness (F_1_ = 2.69, P = 0.10) did not (Fig 3). Pairwise comparisons revealed while fungal α-richness did not differ at Makiki or Palikea, α-richness was elevated on preferred plants at Mt. Ka‘ala (Fig 3). Similarly, α-richness differed across sites (F_2_ = 5.25, P = 0.0069), but diversity (F_2_ = 0.02, P = 0.98) and evenness (F_2_ = 0.45, P = 0.64) did not differ among sites (Fig 3). Makiki had elevated α-richness relative to Palikea, but Mt. Ka‘ala had similar richness to Makiki and Palikea (Fig 3). There were no significant site by preference interactions for fungi (α-richness: F_2_ = 0.91, P = 0.41; diversity: F_2_ = 1.07, P = 0.34; evenness: F_2_ = 1.18, P = 0.30).

### OTU selection

We first list the top ten most important bacterial OTUs (Table 4) and fungal OTUs (Table 5) based on our criteria that incorporates data from multiple sites to identify OTUs that may represent important microbial food resources for arboreal snails. Sequences for these OTUs can be found in supplemental FASTA files (S2, S3). The average relative abundances of the top ten bacteria and fungi identified as potentially important food resources for snails on preferred plants is 2.2%, and 9.3%, respectively (Tables 4, 5). We also report the most abundant bacterial and fungal OTUs on preferred and avoided plants at each site (Figs 4, 5). While lists of the most abundant microbes have been used to characterize microbe-snail encounters [16–19], many bacterial OTUs (e.g., bacteria OTUs 16, 41, and 129) are common on both preferred and avoided plants across sites. None of the most abundant bacterial at our sites were identified using our multi-site criteria that also incorporates relative abundances. In contrast, five of the 10 fungi OTUs identified using our multi-site preference criteria were also one of the top 10 most abundant fungi on preferred plants at a site (Fig 5).

**Table 4:** Selected bacterial OTUs based on preference ratio and abundance on preferred plants at Mt. Ka‘ala, Palikea and Makiki in 2023. We did not calculate variance for the preference ratios because we focused on identifying microbes that consistently had elevated abundances on preferred plants across sites.

OTU ID	Phylum	Family	Preference Ratio			Average Abundance (%)	
			Mt. Ka’ala	Makiki	Palikea	Preferred	Avoided
ed0744a38a060591cd7b77339cc96920	Actinobacteriota	Intrasporangiaceae	0.54	0.07	0.64	0.53	0.26
be7dc20c63379c77044c9ed72b7e08ab	Proteobacteria	Rhodanobacteraceae	0.00	0.83	0.45	0.37	0.05
3156c1dd5f3ae35310c724cc98781e13	Proteobacteria	Sphingomonadaceae	0.52	0.31	0.27	0.27	0.12
0e7cb51785812ab465ac0e2e6179d0b9	Bacteroidota	Microscillaceae	0.94	0.00	1.00	0.23	0.01
ac6646470d60044ffca581d2b7c797bf	Proteobacteria	Beijerinckiaceae	0.86	0.50	0.68	0.23	0.02
8e65f3153acd16042dda032b745855ba	Proteobacteria	Oxalobacteraceae	0.07	0.52	0.71	0.20	0.06
6adaed5f96346cd3bb4b2f814e344822	Bacteroidota	Flavobacteriaceae	1.00	0.06	0.65	0.17	0.13
ea3b0b8b05d1df09f9a032c53c808019	Verrucomicrobiota	Chthoniobacteraceae	0.00	1.00	0.99	0.16	0.00
6fa56f814198f6f55fc192a429aeab7d	Proteobacteria	Rhodanobacteraceae	0.34	0.61	0.41	0.13	0.05
6f1005d82269d8d08b87bafb29012e8d	Bacteroidota	Chitinophagaceae	0.45	0.23	0.73	0.12	0.04

**Table 5:** Selected fungal OTUs based on the preference ratio and the abundance on preferred plants at Mt. Ka‘ala, Palikea and Makiki in 2023. We did not calculate variance for the preference ratios because we focused on identifying microbes that consistently had elevated abundances on preferred plants across sites.

OTU ID	Phylum	Family	Preference Ratio			Average Abundance (%)	
			Mt. Ka'ala	Makiki	Palikea	Preferred	Avoided
aabb97fc9b66c78aa90656285b14f99c	Ascomycota	(C) Dothideomycetes^A^	0.80	0.60	0.00	2.21	0.25
0af21e0127a4e9ec639dc2b421236d6c	Ascomycota	Cladosporiaceae	1.00	0.95	0.00	1.56	0.04
fc6f6e58ea0dde06bf49ff4b7f629436	Ascomycota	(O) Helotiales^B^	1.00	0.00	1.00	1.08	0.00
ba1d4e81c0e89675bbdfe0bc37a792b9	Basidiomycota	Bulleraceae	0.00	0.90	0.57	1.06	0.27
c851ed7b49d30da5966a6a26ccd55131	Ascomycota	Mycosphaerellaceae	0.00	0.81	0.84	0.75	0.08
d2a2f6dfeefa1f6d335ec684678ddb37	Ascomycota	Nectriaceae	0.96	0.36	0.00	0.66	0.04
efab57cebafd2fdc91f3c8c0e50fbcd5	Ascomycota	(O) Chaetothyriales^C^	1.00	0.56	0.35	0.63	0.20
e699fba1da262aef704bc0fdedb24ef1	Ascomycota	Herpotrichiellaceae	0.98	0.00	0.85	0.54	0.04
0445b37605af93a760b2f05aaaeb85d4	Ascomycota	Neodevriesiaceae	0.00	0.89	0.91	0.45	0.02
5481721b356f73b906bad15170eec8f8	Basidiomycota	(C) Microbotryomycetes^C^	0.99	0.00	0.89	0.39	0.01

^A^ This OTU was only classified up to Class Dothideomycetes.

^B^ This OTU was only classified up to Order Helotiales.

^C^ Both of these OTUs are in Incertae sedis of the indicated Order/Class.

**Fig 4:**
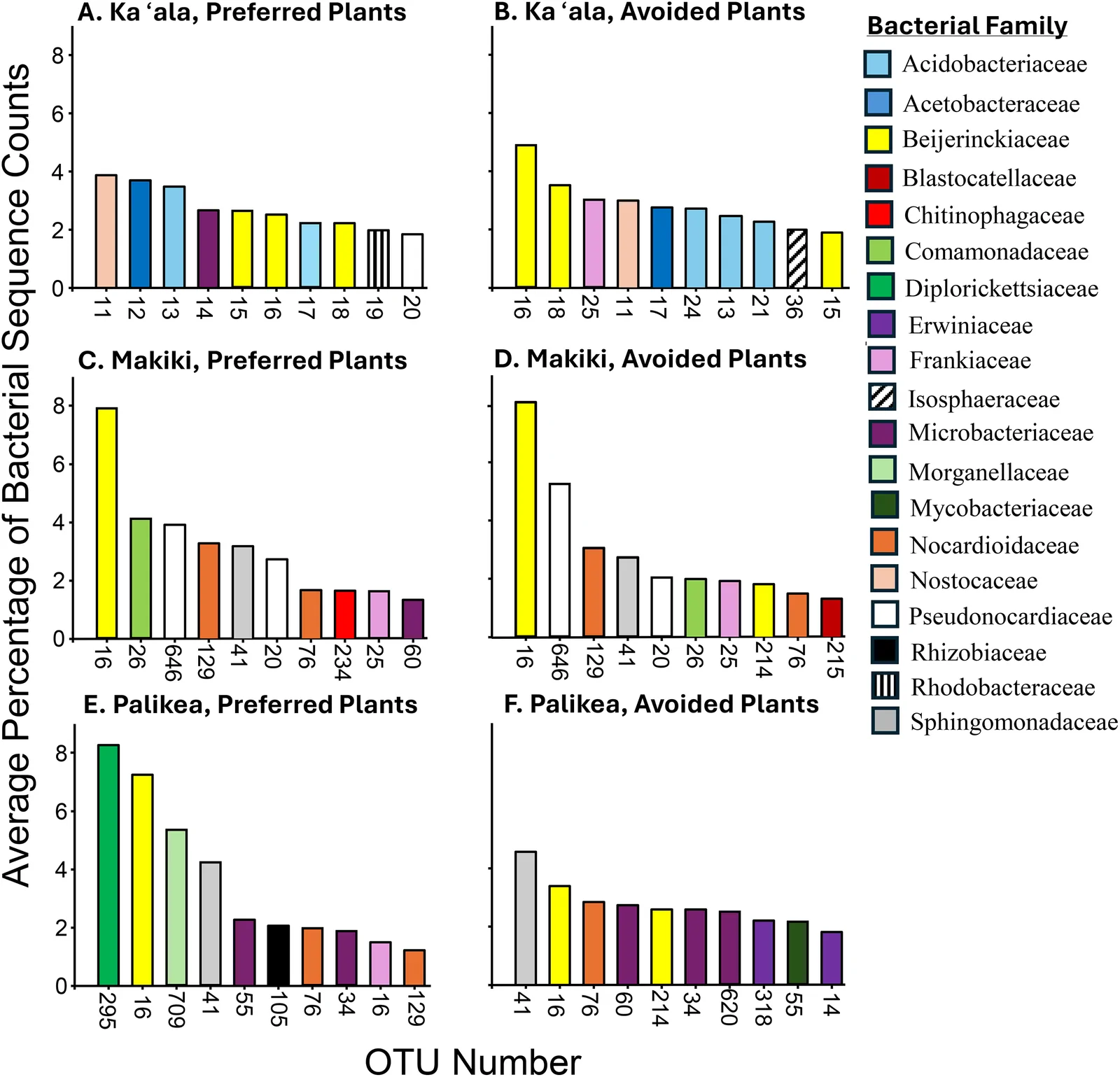
Rank abundance of the 10 most abundant bacterial OTUs on plants preferred and avoided by snails at each site. Different colors highlight OTUs from different families. None of the bacterial OTUs were identified as potentially being important for Hawaiian land snails in Table 4.

**Fig 5:**
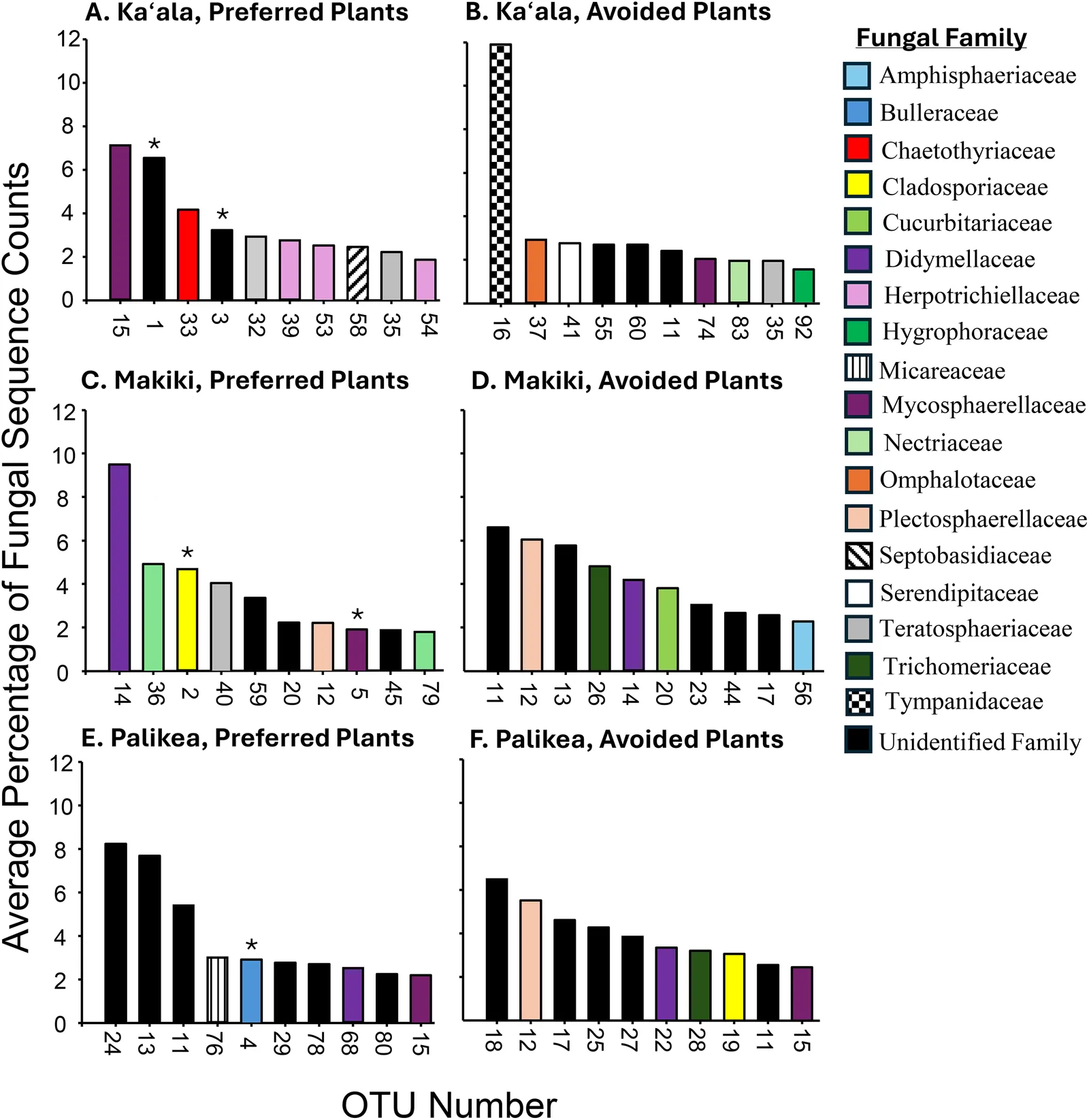
Rank abundance of the 10 most abundant fungal OTUs on plants preferred and avoided by snails at each site. Different colors highlight OTUs from different families. * highlights OTUs that were identified as potentially being important for Hawaiian land snails in Table 5.

## Discussion

This study extends a plant preference–microbiome framework across multiple forest types and years to evaluate the microbial correlates of arboreal snail feeding ecology in Hawaiʻi. Across three sites on Oʻahu, bacterial and fungal assemblages consistently differed between plants preferred and avoided by native snails, supporting the idea that plant selection may be mediated, at least in part, by epiphytic microbial composition rather than plant identity alone. Although site-level differences in microbial assemblages were strong, preference-related patterns remained detectable, demonstrating that this framework is robust to geographic and ecological heterogeneity.

Variation among sites reflects differences in plant communities, snail assemblages, and abiotic conditions, all of which are known to shape phyllosphere microbiomes [30,48–51]. While these biotic and abiotic drivers complicate efforts to identify universal dietary microbes, they underscore an important pragmatic reality, no two sites will have similar plant, snail, or microbial assemblages, and thus the importance of a comparative approach. Rather than seeking identical microbial assemblages across sites or characterizing microbial assemblages that snails encounter at each site, our framework seeks to identify taxa that are repeatedly enriched on preferred plants across sites, as the persistence of enriched microbial taxa on preferred plants may be better at indicating which microbes are meaningful components of snail-associated microbial resources. However, additional laboratory tests are required to determine this.

Using conservative and transparent criteria that incorporate patterns across sites, we identified a limited set of bacterial and fungal OTUs consistently enriched on preferred plants. We feel this approach is better than examining the most common taxa on preferred and avoided plants as many of the most abundant microbial taxa are widespread across sites and common on plants that are both preferred and avoided by Hawaiian arboreal snails. This is especially the case for bacteria as none of the most abundant species at our sites were also identified as important using our multi-site criteria. In contrast, five of the 10 fungal species identified as important using our multi-site criteria were also listed as one of the most abundant fungal OTUs on preferred plants at a site. While both approaches provide different insights into how snails may interact with the microbial assemblages on plants, we feel a multi-site approach provides key insights into which microbes may be important for a variety of arboreal snail species. However, it is important to note that our analyses are intended to develop hypotheses about which microbes are important, and findings are not presented here as confirmed dietary components, but as experimentally tractable candidates that should be further evaluated in laboratory trials [see 18,26–27 for different experimental approaches]. Together, these findings narrow the scope of future feeding trials and provide the foundation for well-formed hypotheses in these feeding trials (e.g., allowing for *a-priori* hypotheses about which microbes snails may prefer and/or avoid) improving the experimental design of studies that aim to understand threatened and endangered Hawaiian arboreal snail diets.

Several bacterial and fungal OTUs identified via our multi-site criteria belong to lineages known to influence plant physiology, stress tolerance, or macro-invertebrate dietary availability [18–20,52–62]. Among the bacteria OTUs demonstrating elevated relative abundances on preferred plant species across our O‘ahu study sites, members of the Sphingomonadaceae (Table 4) are recognized as core, highly consistent colonizers of the wild plant phyllosphere [52]. These taxa provide critical structural stability to leaf surface biofilms, mitigate host plant stress, and actively exclude foliar pathogens [52]. Their elevated abundance on preferred plants may indicate that arboreal snails target these ubiquitous, non-pathogenic bacterial layers while grazing. Bacterial OTUs belonging to the Intrasporangiaceae and Beijerinckiaceae also appear as highly enriched on preferred plants (Table 4). While the Intrasporangiaceae are predominantly associated with studies of soil and wastewater systems, recent metagenomic insights have revealed that they act as vital foliar endophytes and epiphytes that plants actively recruit under environmental stress [53]. These bacteria act as a functional barrier that protects the host foliage against environmental and osmotic fluctuations. The Beijerinckiaceae are notable nitrogen-fixing methylotrophs that utilize methanol byproducts generated during plant leaf growth, directly altering the nutritional chemistry of the phyllosphere [54]. The Rhodanobacteraceae was also among those with high relative density and enhanced abundance on preferred plants across sites (Table 4). Members of this group of bacteria are increasingly recognized as key functional components of the healthy plant phyllosphere, where they participate in volatile organic compound transformation and the modulation of local microbial community dynamics [55]. By altering the metabolic profile of the leaf surface, these bacterial assemblages can influence the localized chemical cues that arboreal snails may utilize to navigate toward preferred foliage. Furthermore, Sphingobacteriaceae represented a dominant component among our top preference-ranked bacterial taxa (Table 4). This family is of particular significance to malacological ecology, as members of the Sphingobacteriales have been previously documented as major structural components of both the wild host-plant phyllosphere and the fecal microbiomes of arboreal Hawaiian land snails, such as *Achatinella mustelina* [17]. The consistent enrichment of Sphingobacteriaceae on preferred plants reinforces the hypothesis that these snails may be targeting highly predictable, functional bacterial biofilms that are adapted to thrive on specific native foliage. Because these bacteria are robust, slime-producing colonizers, they likely provide a substantial and easily accessible caloric and polysaccharide resource for grazing gastropods. These highly functional bacterial OTUs on the leaf-surface serve a multi-tiered ecological purpose, acting simultaneously as a possible beneficial nutritional reservoir for grazing arboreal snails and as a vital physiological component of healthy host-plant communities. Thus, playing foundational roles in maintaining the overall health and stability of native Hawaiian plant communities.

Parallel trends were observed within the fungal communities. Crucially, a distinct Cladosporiaceae OTU (Table 5) displayed enrichment on preferred plants (1.56%) relative to avoided plants (0.04%) across our study sites. *Cladosporium* spp. are considered ecologically necessary in Hawaiian malacological conservation, as they have been widely deployed as a dietary supplement to stabilize fecundity and growth in captive-rearing initiatives for threatened tree snails [17,18,26]. This OTU’s presence on our lists lends empirical support to its role as a valid candidate dietary component. The enrichment of this Cladosporiaceae OTU on preferred plants in our study reflects ecological dynamics extending beyond the Hawaiian archipelago. Worldwide, *Cladosporium* species are recognized as fundamental nutritional resources for diverse grazing invertebrates and microarthropods due to their highly palatable structural composition and dense lipid storage capabilities [56–58]. Concurrently, members of the Cladosporiaceae actively contribute to host-plant health. Global isolates from healthy wild foliage demonstrate that commensal *Cladosporium* strains can synthesize growth-promoting phytohormones like gibberellins [59]. Similar to patterns seen with regard to bacterial assemblages on preferred plants, snail preferences for plants harboring elevated *Cladosporium* biomass may represent a nutritionally dense fungal biofilm that simultaneously optimizes host-plant vigor.

However, interpreting the importance of various species in the Cladosporiaceae is difficult. While Meyer et al. [21] found that *Cladosporium* spp. were often absent or rare in their samples from Mt. Kaʻala, this study found that some Cladosporiaceae OTUs may be important for Hawaiian land snails. Additionally, O’Rorke et al. [18] found that *Cladosporium* sp. was one of three, equally preferred food sources in feeding trials with *Auriculella diaphana*. The authors also found that *Cladosporium* sp. used to supplement diet for captively reared snails was the sixth most common species for snail diet in the wild based on their surveying techniques [18]. However, while one Cladosporiaceae OTU was also found to be one of the most abundant fungi on preferred plants at Makiki, other Cladosporiaceae OTUs have higher relative abundances on avoided plants at other sites. This suggests that differences among microbes within families at the OTU level may have important ecological implications for snails. In captive breeding studies, a diet of native vegetation supplemented with *Cladosporium* sp. has been shown to improve fecundity and survivorship with vegetation, but *Cladosporium* sp. on their own are insufficient [26]. Taken together, these results suggest that we should continue to evaluate various *Cladosporium* species to understand the complexity of interactions involved in Hawaiian arboreal snail diets. Specifically, it would be interesting to know if the *Cladosporium* sp. used in captive rearing is the same OTU as the one we identified as important, or if it is a different species, could rearing efforts be improved with this, or additional Cladosporiaceae OTUs. These complex dynamics emphasize the importance of studies into arboreal snail diets to improve captive rearing efforts, especially as many threatened and endangered snail diets are now predominately supplemented with *Cladosporium* sp. [18,20,26].

We also noted the high presence of Herpotrichiellaceae and other Chaetothyriales on preferred flora (Table 5). These lineages are characterized as microcolonial black yeasts that synthesize heavily melanized cellular matrices [60,61]. This may facilitate structural resilience against extreme desiccation and intense solar ultraviolet exposure on the plant cuticle [62], thereby providing a highly stable, dependable biofilm biomass for grazing arboreal gastropods.

Interannual analyses at Mt. Kaʻala revealed that both bacterial and fungal assemblages in June 2023 differed from those collected in November 2020. Assemblages from earlier years using OTUs did not differ from one another. While we did not find differences between July 2018 and November 2020 at the OTU level, Meyer et al. [21] did find differences in fungal assemblages between those two sampling events at the ASV level. These results indicate temporal variability in epiphytic microbial communities but also show that differences between preferred and avoided plants remain consistent despite year-to-year variation. While multiple factors could contribute to interannual shifts, including climate variability and natural microbial turnover, these results emphasize the importance of repeated sampling and caution against assuming temporal stability in microbial resources. Differences in microbial samples collected in November 2020 (wet season) and June 2023 (dry season) in our study, and differences in fungal assemblages between samples collected in July 2018 (dry season) and November 2020 (wet season) in the Meyer et al. [21] study, suggest that there may be subtle seasonal effects on microbial assemblage structure that should be incorporated into future monitoring of microbial assemblages. Establishing this baseline provides us with the opportunity to better explore how microbial assemblages are changing which is key to understanding the effects of climate change, habitat modifications, and other disturbances, and how these factors may impact snail fitness and forest health. Once we have a better understanding of which microbes constitute Hawaiian arboreal snail diets, we will be better suited to: (1) answer questions related to habitat quality for threatened Hawaiian snails, (2) explore how disturbances, like changing climates, may influence habitat quality, and (3) provide informed restoration and monitoring recommendations to preserve habitats that can support these threatened snail species.

We found little evidence that snails preferentially select plants with higher microbial richness or diversity. With the exception of elevated bacterial and fungal richness on preferred plants at Mt. Kaʻala, alpha diversity metrics did not differ between preferred and avoided plants. This pattern suggests that specific microbial taxa or assemblages, rather than overall diversity, are more likely to influence snail feeding behavior.

## Conclusion

Our multi-site comparative approach that identifies microbes enriched on vegetation preferred by snails provides an empirical foundation for the identification of candidate microbial taxa that may be important dietary components for threatened and endangered Hawaiian snails. Identification of candidate microbes will improve hypotheses development and the experimental design of feeding experiments aimed at improving threatened and endangered snail captive-rearing initiatives. By moving beyond a narrow reliance on a small number of readily culturable fungi, this framework allows conservation practitioners to focus culturing efforts on species that may have an elevated chance of improving snail fitness and design dietary protocols that replicate more ecologically representative and nutritionally complete microbial biofilms.

Importantly, this study does not argue that individual microbial taxa alone dictate snail diet, plant selection, or fitness. Controlled laboratory validation remains an essential next step to confirm the precise nutritional contributions of these target microbes, and to determine which bacterial and fungal taxa and assemblages better support snail growth, fecundity, and long-term survivorship [16,18,26,27]. Active efforts to culture individual candidates, including key members of the Sphingomonadaceae, Beijerinckiaceae, and specific Cladosporiaceae variants, are currently underway. However, the inherent difficulty in isolating and culturing targeted microbial epiphytes and endophytes remains a major technical bottleneck. Until these candidate microbes can be reliably isolated and mass-propagated, molecular-guided microbial assemblage profiles offer important insights for interpreting wild dietary habits and efforts to engineer complex synthetic biofilms that may improve snail fitness.

By linking macro-scale plant preference data with micro-scale microbial community analyses, this study advances our fundamental understanding of arboreal snail ecology and provides a path forward for embedding microbiome diagnostics into applied conservation decision-making. As habitat degradation, invasive species, and climate change continue to reshape native Hawaiian forests, conservation tools that identify and preserve important microbial epiphytes, which are critical for forest health and form the foundational components of snail diets, will become increasingly critical. The framework presented here offers a reproducible starting point, successfully bridging the gaps between field ecology, high-resolution sequencing and bioinformatics, and endangered species management.

## Supporting information

S1 File Zip-file including the sample by OTU matrices for the both bacteria and fungi. (ZIP)

S2 File Zip-file including sequences for each of the ten bacterial and fungal OTUs identified by using our multi-site preference analysis identified by their OTU identifier from Tables 4 and 5. (ZIP)
